# The emerging role of intraoperative nanopore sequencing on the neurosurgical strategy in paediatric embryonal brain tumours

**DOI:** 10.1007/s00381-026-07377-8

**Published:** 2026-07-03

**Authors:** Oscar H. J. Eelkman Rooda, Mariska Sie, Sabine L. A. Plasschaert, Mariëtte E. G. Kranendonk, Eelco W. Hoving

**Affiliations:** 1 Princess Máxima Center for Pediatric Oncology, PO box 113, 3720 AC Bilthoven, Utrecht, The Netherlands; 2 Department of Neurosurgery, Erasmus MC - Sophia Children’s Hospital, Rotterdam, Netherlands

**Keywords:** Intraoperative molecular diagnostics, Nanopore sequencing, Paediatric embryonal brain tumours, Surgical decision-making, Extent of resection, Medulloblastoma

## Abstract

Paediatric neurosurgeons have traditionally relied on preoperative imaging and intraoperative frozen section pathology to guide surgical decision-making in paediatric embryonal brain tumours. However, substantial radiological overlap between medulloblastoma, atypical teratoid/rhabdoid tumour (AT/RT) and embryonal tumour with multilayered rosettes (ETMR) often creates diagnostic uncertainty to support the optimal strategy concerning the balance between the oncological need of radical resection and a maximal safe resection in order to avoid neurological morbidity. For this *Child’s Nervous System* special collection on paediatric embryonal tumours, we present our experience with the potential role of intraoperative real-time molecular diagnostics (‘nanopore sequencing’) to address this innovation with the potential to inform surgical decision-making. Specifically, we describe the aspects of workflow implementation, integration with neuropathology and surgical decision dynamics, and we illustrate how intraoperative molecular classification can influence surgical decision-making. Particular attention is given to discrimination of subgroup-specific identification in medulloblastoma, AT/RT and ETMR, tumour entities in which real-time confirmation of aggressive biology may support better informed decisions regarding extent of resection. Finally, we discuss key unresolved questions and outline priorities for future clinical and translational studies aimed at defining the impact of intraoperative nanopore sequencing on surgical morbidity, extent of resection and long-term outcomes.

## Introduction

Embryonal brain tumours are the most common malignant CNS tumours in children and, despite effective multimodal therapies, they remain among the leading causes of cancer-related mortality in children [1, 2]. Primary treatment remains surgical resection, during which a constant balance between maximal tumour removal and risk of neurological morbidity must be maintained. The optimal extent of resection (EoR) is increasingly individualised, depending on tumour biology, disease stage, patient age and the availability of effective adjuvant therapy where the ultimate goal is achieving survival with an acceptable quality of life as the preferred outcome.

Importantly, the oncological relevance of EoR is not uniform across embryonal tumours or their molecular subgroups. In medulloblastoma, retrospective analyses suggest that the survival impact of gross total resection (GTR) differs between WNT, SHH, Group 3 and Group 4 tumours, but this has not been prospectively evaluated [3]. Consequently, any potential advantage of more radical resection must be interpreted cautiously and weighed against the risk of neurological morbidity [3]. These uncertainties highlight that surgical strategy can only be meaningfully optimised when biological tumour identity is known at the time of decision-making.

A recurring challenge in the paediatric neurosurgical literature is the reliance of the term “maximal safe resection”, a concept that is widely accepted, intuitively appealing, yet inherently variable in its practical interpretation. The definition of “safe” resection varies between surgeons, levels of experience, institutions, and healthcare contexts and is often influenced by implicit value judgements regarding neurological risk, long-term disability and anticipated survival benefits. Many studies evaluating extent of resection focus primarily on radiographic or survival endpoints, while patient-reported outcome measures are frequently absent. Consequently, recommendations for surgical strategies concerning radicality are often generalised rather than being based on combined aspects of survival and functional outcomes.

Nevertheless, over the past decades, paediatric neurosurgery has steadily become safer. Improved anaesthetic techniques and specific neurosurgical advances such as the use of intraoperative ultrasound (io-US), neuronavigation, intraoperative neuromonitoring (IONM) and more recently intraoperative MRI (io-MRI) have contributed to substantial reductions in neurological morbidity by providing improved anatomical orientation and sustaining functional preservation [4, 5]. However, while these modalities inform surgeons *where* they operate, they provide limited insight into *what* biological entity is being resected.

This limitation is particularly relevant for embryonal tumours. Preoperative MRI is often inconclusive, and radiological distinction between the various differential diagnoses of AT/RT, medulloblastoma and ETMR is not always feasible [6, 7]. Intraoperative frozen section analysis of tumour tissue aids diagnosis and has long been constituted as a key determinant in guiding intraoperative decision-making. However, it typically provides only broad tumour type categories such as ‘embryonal tumour’ and its accuracy is highly dependent on the expertise of the pathologist [8].

On top of that, the final tumour classification increasingly relies on molecular characteristics and frequently becomes available days till weeks postoperatively when it can no longer influence intraoperative decisions regarding EoR. This information gap represents a central limitation of current practice.

Recent developments in real-time molecular methylation-based diagnostics using intraoperative nanopore sequencing technology provide additional crucial objective information about the tumour during resection. Sequencing platforms can now generate low-coverage methylation and copy-number profiles within 30–90 min, enabling AI-assisted tumour classification while surgical planning is still adaptable to decide for the optimal surgical strategy concerning extent of resection in the final phase of the tumour surgery [9, 10]. Several groups have demonstrated the feasibility and diagnostic accuracy of intraoperative nanopore sequencing and early implementation studies suggest that real-time molecular diagnosis guides surgical strategies and alters these strategies in approximately 10–20% of cases [11–13]. Importantly, this technology may support neurosurgeons with additional biological information during intraoperative decisions, but also creates unique opportunities to systematically study the definite, biology-specific impact of EoR in a prospective and individualised manner.

Concerning this special collection on paediatric embryonal tumours, we present our experience of current evidence on intraoperative nanopore sequencing and its emerging impact on neurosurgical strategy. We focus on three interrelated aspects: (1) implementation challenges and workflow redesign; (2) early evidence demonstrating strategy adjustments, with detailed information on medulloblastoma subgroups, AT/RT, and ETMR and (3) key unresolved questions and essential/relevant future studies required to define and validate this emerging paradigm and its clinical value. Across these sections, we explicitly address what constitutes oncological optimal resection in different biological contexts and how real-time molecular information can support intraoperative judgements.

## Implementing real-time sequencing in the operating room

### Workflow redesign

The introduction of intraoperative nanopore sequencing requires re-establishing diagnostic workflows. Generally, routine molecular testing relies on formalin-fixed tissue and batch-based processing, whereas nanopore sequencing requires fresh tumour material and rapid handling during surgery. To ensure that molecular analysis is performed on representative tumour tissue, early adopters have integrated sequencing into the intraoperative diagnostic pathway at the time of frozen section analysis, using a split-sample approach in which tumour tissue is processed for frozen section analysis and DNA extraction in parallel, followed by library prep and nanopore sequencing [10].

In our workflow, intraoperative nanopore sequencing is not intended to replace neuropathological expertise, but to complement frozen section analysis with sequencing-based tumour classification enabling an integrated intraoperative histomolecular diagnosis that supports more confident decision-making regarding the EoR. This “human-in-the-loop” (pathologist) approach ensures that molecular classifiers only contribute information for benefit of proper surgical decision-making (2nd human; neurosurgeon), aligning with broader principles for responsible clinical implementation of artificial intelligence-based diagnostics [14].

Once these elements are established, intraoperative sequencing has proven feasible and easy to integrate into neurosurgical tumour resections. This intraoperative histomolecular tumour diagnosis provides the neurosurgeon with new information to choose the proper resection strategy in the individual patient being operated.

In centres with limited access to specialised neuropathological expertise, nanopore sequencing may have an even greater clinical impact as it provides detailed and objective diagnostic information. However, the results still need to be interpreted in the clinical and radiological context. Multiple institutions have now demonstrated sustainable intraoperative sequencing workflows with lead times under 90 min and high concordance with final molecular diagnoses [9–12].

These experiences collectively indicate that real-time molecular diagnostics can be integrated into routine neurosurgical practice without disrupting operative flow, provided that workflows are carefully designed and interdisciplinary communication is robust.

### Neurosurgical timing and decision dynamics

A critical aspect of workflow redesign concerns timing. Neurosurgeons rarely halt a procedure while awaiting diagnostic information. Instead, tumour resection typically proceeds while molecular/frozen section analysis runs in parallel. The practical objective is therefore not to deliver a diagnosis as early as possible, but to ensure that actionable information becomes available before the surgeon reaches anatomically or functionally critical boundaries.

In our experience, sending tissue as soon as the tumour is exposed ensures that results are available well before the surgeon reaches potentially anatomically or functionally critical margins. In practice, this timing can fundamentally benefit the decision-making process of the neurosurgeon to choose the appropriate strategy of being radical or more conservative, in this crucial final phase. Decisions that previously relied on probabilistic reasoning based on imaging and equivocal frozen section results can now be enriched by a histo-molecular tumour tissue diagnosis. For the surgeon, the difference between a tentative histological impression and a molecularly supported classification is substantial, particularly in tumour entities where the benefit of gross total resection is highly dependent on the specific diagnosis and patient context. In such settings, real-time molecular information may support a more deliberate balance between oncological intent and neurological risk. Importantly, this paradigm also creates an opportunity to systematically study whether biologically informed moderation translates into reduced complications (such as cerebellar mutism) within prospective research studies.

### Cost and feasibility considerations

Sequencing is performed on portable devices such as the MinION Mk1C or PromethION, chosen according to institutional goals (single-sample intraoperative pipelines versus high-throughput sequencing). Basecalling, methylation calling and copy number inference occur in real-time, and machine learning classifiers trained on extensive reference methylomes (Sturgeon, MNP-Nano) typically achieve confident tumour identification within 20–40 min of sequencing [15, 16].

From a health-economic perspective, intraoperative nanopore sequencing has a relatively low barrier to entry when compared with other intraoperative technologies routinely used in neurosurgery (such as MRI, navigation, ultrasound or others). Using a MinION-based workflow, initial setup costs are modest (~ $3000 to acquire a MinION, excluding laptop and basic accessories), while consumable costs are primarily driven by flow cells and preparation, resulting in an estimated per-case cost of approximately $700–$1200. This workflow can be performed using standard computing infrastructure, limiting additional hardware requirements. Implementation does require coordinated collaboration between neurosurgery, neuropathology and molecular laboratory personnel to ensure representative tissue sampling, rapid DNA extraction and integrated interpretation of results. If these costs will outweigh against potential benefits from reducing diagnostic uncertainty, preventing second-look surgeries and enabling earlier histomolecular treatment planning during the initial admission, needs to be clarified.

Beyond high-resource settings, the relatively low capital and infrastructural requirements of nanopore sequencing also create a compelling opportunity to expand access to high-quality molecular diagnostics in low- and middle-income countries, where the majority of the world’s children reside. From this global health perspective, a portable, cost-contained platform capable of delivering detailed molecular information may help reduce global disparities in paediatric neuro-oncological care.

An additional consideration is the potential consolidation of molecular diagnostics. Institutions currently relying on array-based methylation profiling, targeted sequencing panels or whole-exome sequencing may ultimately transition toward nanopore-based platforms as a unified solution. In such a scenario, rapid intraoperative classification could be followed by generation of a comprehensive molecular profile within 24 h [10, 12]. These possibilities underscore the need for prospective health-economic evaluations embedded within clinical implementation studies.

### How real-time molecular diagnostics are changing surgical strategy

#### Tumour-specific surgical implications

Although the 2021 WHO classification defines different subcategories of embryonal tumours, we focus here on the three most clinically consequential: medulloblastoma, AT/RT and ETMR. For each, EoR remains important, albeit to varying degrees, while EoR is prognostic in some entities and subgroups, while in others marginal gains may be small relative to potential morbidity.

#### Medulloblastoma: subgroup-specific surgical strategy

Medulloblastoma comprises four principal molecular groups (WNT, SHH, Group 3 and Group 4) each associated with distinct biology, risk stratification and therapeutic pathways [17, 18]. While achieving a gross total resection (GTR, no visible remnant) or near-total resection (NTR, < 1.5 cm^2^ residual tumour) is generally associated with better outcomes than leaving a larger residual (> 1.5 cm^2^) disease, these distinctions are largely based on retrospective radiological thresholds that are difficult to assess intraoperatively and may not reflect a biologically meaningful boundary [3, 19, 20]. Consequently, the prognostic impact of extent of resection appears context-dependent rather than uniform across molecular subgroups.

For *WNT-activated medulloblastoma*, given its excellent prognosis and sensitivity to adjuvant therapy, it is reasonable to question whether one should refrain from GTR and prevent neurological risk. Intraoperative identification of a WNT tumour therefore supports a surgical strategy prioritising functional preservation once a near-total resection has been achieved.

*SHH-activated medulloblastomas* display marked heterogeneity across age groups. Infants typically receive radiation-sparing regimens; for them, achieving a maximal safe resection may meaningfully influence outcomes, although the independent prognostic value of EoR remains uncertain and is not incorporated into current risk schemes. Moreover, infant SHH tumour may exhibit diffuse or Lhermitte-Duclos-like growth patterns, in which complete resection is technically not feasible. Conversely, evidence suggests that subtotal resection confers only a modest, nonsignificant increase in mortality risk in SHH tumours, raising the possibility that aggressive resection of adherent, high-risk residue may not always be justified. Importantly, other prognostic factors, such as metastatic status, histological features and TP53 mutation carry substantially greater prognostic weight than EoR, yet are largely unavailable intraoperatively. While TP53 status cannot currently be determined in real-time, future rapid sequencing approaches may allow this information to be integrated into surgical decision-making. Until then, real-time molecular identification allows surgeons to integrate subgroup-level biological context into intraoperative decision-making, supporting a more individualised balance between oncological intent and neurological risk.

For *group 3 medulloblastoma*, including MYC-amplified cases, prognosis remains poor despite intensive therapy. Although some series suggest improved outcomes following gross total resection, one of the largest retrospective analyses demonstrates that the effect size of extent of resection might be limited [3]. While intensified adjuvant therapy or subgroup-tailored de-escalation strategies may emerge, for now the evidence does not conclusively support high-risk incremental resection based solely on subgroup 3 identity. Real-time molecular diagnosis enables this deliberation during surgery.

For *group 4 medulloblastomas*, there is a more ambiguous scenario. Although studies have not reached statistical significance, several analyses suggest a trend toward improved outcomes with GTR in group 4 tumours [3]. In our view, this remains the subgroup where intraoperative confirmation may most plausibly tip the balance toward maximal resection, provided it can be achieved safely.

Across subgroups, consensus remains that maximal safe resection is desirable; nevertheless, age, tumour location, disease stage and individual risk tolerance shape the final surgical decision. Real-time molecular identification adds clinically relevant precision to this balancing act. It should be acknowledged though, that the current evidence base for linking EoR to outcome in this specific subgroup of embryonal tumours remains limited, which in turn limits how definitively intraoperative molecular results can guide surgical radicality. Additionally, current risk stratification for medulloblastoma depends on factors beyond molecular subgroup such as histopathological features (e.g., large cell/anaplastic morphology), MYC or MYCN amplification, TP53 mutation (in SHH tumours), and the presence of metastases. These high-risk features often carry significant prognostic weight (sometimes exceeding that of subgroup alone), yet none are yet identifiable via the limited sequencing data available intraoperatively.

Moreover, commonly used thresholds such as a residual tumour area of 1.5 cm^2^ originate from retrospective analyses and represent pragmatic, post hoc cut-offs rather than biologically and surgically meaningful boundaries. In practice, such distinctions are difficult, if not impossible, to assess intraoperatively. Against this backdrop, real-time molecular classification should be viewed not as a definitive arbiter of resection extent in medulloblastoma, but as a tool that enables more informed surgical reasoning in a context where evidence remains incomplete.

### AT/RT: high-malignant biology requiring maximal resection

AT/RT, defined by SMARCB1/INI1 loss, is characterized by fulminant progression and poor survival [21]. Multiple studies demonstrate a strong association between GTR and improved outcomes, supporting a surgical strategy that aims for maximal safe resection with a strong preference for radical tumour removal whenever feasible [22–24].

At the same time, AT/RT does not represent a uniform clinical entity. Outcome can vary substantially with age and tumour location. Although molecular and anatomical heterogeneity within AT/RT has been described, current evidence does not yet support subtype-specific intraoperative surgical strategies. Rather than dictating a fixed surgical decision, the diagnosis of AT/RT informs a risk profile within which surgical decisions must be individualised.

Nevertheless, this underscores the need for intraoperative diagnostics. Frozen section diagnosis of AT/RT is particularly challenging due to its morphological overlap with other embryonal tumours [25]. As a result, intraoperative diagnostic uncertainty may inadvertently temper surgical radicality in a tumour entity where maximal resection is most clearly indicated, even in anatomically challenging regions. In this setting, intraoperative molecular diagnostics serve as an enabling technology that supports a surgical decision process in which maximal resection is pursued when the anticipated oncological benefit justifies the surgical risk.

### Pineoblastoma and ETMR: rare embryonal tumours where extent of resection matters

Pineoblastoma represents a rare embryonal tumour in which extent of resection appears to influence outcome [26, 27]. Given the deep midline location and proximity to critical venous and midbrainstem structures, surgical decision-making in pineoblastoma is often finely balanced. In this context, reliable intraoperative tumour identification may support more informed judgement whether additional resection is justified, rather than driven by anatomical feasibility alone.

ETMR, defined by C19MC amplification and multilayered rosettes, is among the most aggressive paediatric CNS tumours [2, 28]. Median survival remains under two years [29], and the few long-term survivors nearly all underwent complete resection followed by intensive chemotherapy [30, 31].

The feasibility and oncological relevance of complete resections are strongly influenced by tumour location. ETMRs may arise supratentorially, in the posterior fossa, or (more rarely) within the brainstem, where radical resection is inherently unsafe or impossible.

At diagnosis, approximately 20% of patients present with metastatic disease, and treatment protocols frequently incorporate focal radiotherapy. However, craniospinal irradiation is often not feasible due to the very young age of affected patients and local disease control through surgery may carry disproportionate importance when anatomically achievable.

Given its rarity and overlapping radiological and histological features, ETMR is frequently not recognised intraoperatively which might lead to a missed opportunity for maximal resection in surgically accessible locations. In this specific context, real-time molecular diagnostics align surgical decision-making with the biological imperative of the disease.

### Illustrative intraoperative decision-making scenarios

**Fig. 1:**
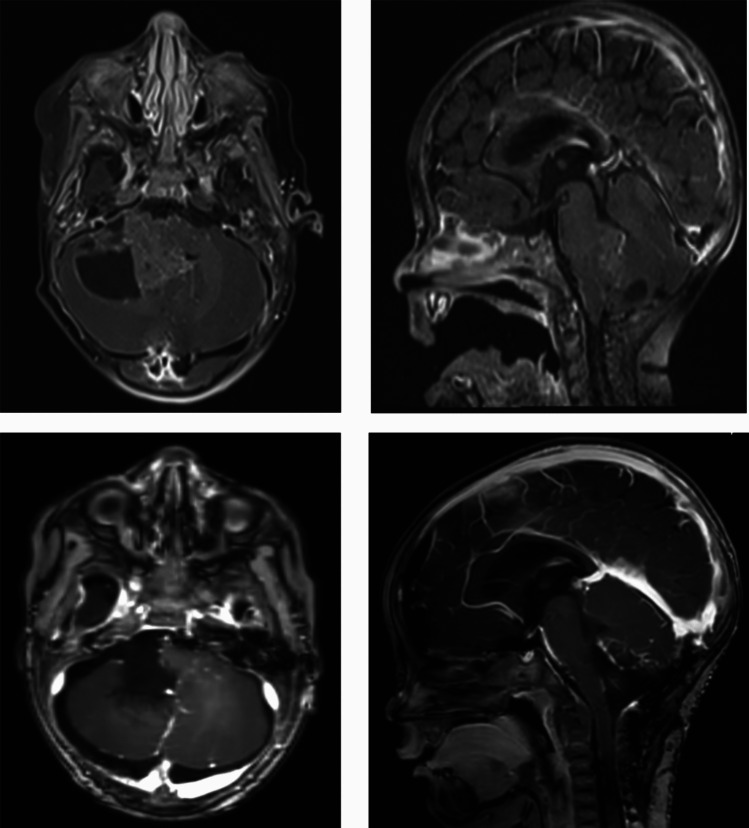
Pre- and postoperative magnetic resonance imaging of a fourth ventricular tumour illustrating preoperative diagnostic uncertainty. (Top panels) Preoperative axial and sagittal T1-weighted contrast-enhanced MRI demonstrating a midline fourth ventricular mass with imaging characteristics compatible with a medulloblastoma, but with overlapping features that do not exclude alternative embryonal entities such as atypical teratoid/rhabdoid tumour (ATRT). (Bottom panels) Intraoperative MRI showing near-complete resection with no visible residual tumour. This case highlights the importance of integrating intraoperative diagnostic information when determining the appropriate extent of resection

Here, we present a case illustrating how intraoperative molecular diagnostics may support surgical strategy in the setting of diagnostic uncertainty (Fig. 1). Preoperatively, imaging raised suspicion for an embryonal tumour, but could not reliably distinguish between medulloblastoma and AT/RT.

Intraoperative frozen section analysis reported an embryonal tumour with a preference for AT/RT, while a large cell anaplastic (LCA) medulloblastoma could not be excluded. In this context of persistent diagnostic ambiguity, real-time nanopore sequencing provided molecular information consistent with AT/RT. Although some surgeons may have pursued maximal resection based on imaging and frozen section findings alone, the integrated histomolecular diagnosis substantially increased intraoperative diagnostic confidence at the moment of final surgical decision-making. In this case, molecular confirmation reduced residual uncertainty and supported continuation toward maximal safe resection. In our opinion, this case exemplifies how intraoperative nanopore sequencing can resolve clinically meaningful diagnostic uncertainty and support justified surgical decisions in real time, beyond what imaging and frozen section analysis alone can provide.

## Future outlook: how to go to clinical translation

The rapid adoption of intraoperative nanopore sequencing raises an important methodological concern in which clinical implementation outpaces generation of high-level evidence. As with prior technologies, the window for conducting a well-controlled prospective trial may close quickly once the technology becomes regarded as “standard”, necessitating alternative study designs.

Prospective multicentre cohort studies using harmonised workflows and propensity-matched comparators may therefore represent the most feasible next step. Such studies should focus not only on extent of resection, but also on neurological morbidity, need for second-look surgery, time to adjuvant therapy and patient-reported outcomes.

Importantly, the assumption that earlier initiation of adjuvant therapy necessarily improves outcome warrants careful scrutiny, underscoring the need to integrate survival metrics with functional and quality-of-life endpoints. Therefore, last but not least, embedding structured patient-reported outcomes into surgical trials will be essential to translate ‘maximal safe resection’ from a technical principle into a patient-centred construct.

Complementary approaches include stepped-wedge implementation trials, entity-focused prospective registries for AT/RT and ETMR and decision-impact studies that document intended surgical strategy versus the actual performed surgical strategy. Comprehensive health-economic evaluations should accompany these efforts, incorporating morbidity and long-term quality-of-life effects. Together, these approaches will determine whether intraoperative nanopore sequencing evolves from a promising innovation into a fundamental component of biologically informed paediatric oncological neurosurgery.

## Conclusion

Intraoperative molecular diagnostics may represent an important step in paediatric neurosurgery. By resolving tumour identity during the surgery, nanopore sequencing allows neurosurgeons to align the extent of resection with biological necessity. This may support escalation of resection in AT/RT and ETMR, moderating it in WNT medulloblastoma, and refining strategy across the molecular spectrum. Early evidence shows that intraoperative molecular data reshape surgical decision-making in a meaningful minority of cases and prevent diagnostic uncertainty in many more.

The coming decade will determine whether this paradigm becomes standard practice. Rather than uniformly reducing morbidity or improving survival, the integration of surgical precision with molecular identification has the potential to better individualise neurosurgical decision-making, aligning operative risk with biological prognosis. When embedded within prospective studies that capture neurological, oncological and patient-reported outcomes, this approach may ultimately support durable survival with acceptable quality of life and redefine paediatric brain tumour surgery as a genuinely biology-driven discipline.

## Data Availability

No datasets were generated or analysed during the current study.
